# Assessing the Acceptability and Usability of an Interactive Serious Game in Aiding Treatment Decisions for Patients with Localized Prostate Cancer

**DOI:** 10.2196/jmir.1519

**Published:** 2011-01-12

**Authors:** Lindsey Reichlin, Nithya Mani, Kara McArthur, Amy M Harris, Nithin Rajan, Clifford C Dacso

**Affiliations:** ^4^School of International and Public AffairsColumbia UniversityNew York, NYUnited States; ^3^University of Texas Southwestern Medical SchoolDallas, TXUnited States; ^2^Bauer College of BusinessUniversity of HoustonHouston, TXUnited States; ^1^The Abramson Center for the Future of Health, a joint effort of The Methodist Hospital Research Institute and College of TechnologyHouston, TXUnited States

**Keywords:** serious games, prostate cancer, shared decision-making, usability

## Abstract

**Background:**

Men diagnosed with localized prostate cancer face a potentially life-altering treatment decision that can be overwhelming. Enhancing patient knowledge through education can significantly reduce feelings of uncertainty while simultaneously increasing confidence in decision making. Serious games have been shown in other populations to increase health knowledge and assist with the health decision-making process. We developed an interactive serious game, Time After Time, which translates evidence-based treatment outcome data into an accessible and understandable format that men can utilize in their prostate cancer treatment decision-making process. The game specifically aims to raise men’s awareness and understanding of the impact of health-related quality of life issues associated with the major treatment options and to enrich their conversations with their health care providers.

**Objective:**

This study determined the acceptability and usability of the alpha version of Time After Time, an interactive decision aid for men diagnosed with localized prostate cancer, in order to inform future iterations of the serious game.

**Methods:**

The study employed a mixed methods approach to assess the acceptability and usability of the Time After Time serious game using qualitative focus groups and a quantitative Likert scale survey.

**Results:**

A total of 13 men who had already completed treatment for localized prostate cancer completed the survey and participated in focus group meetings. The majority of the study participants rated Time After Time as an appropriate decision tool for localized prostate cancer and verified that it meets its goals of increasing focus on side effects and generating questions for the patient’s health care team. However, participants also expressed concerns about game usability and the diversity of information covered regarding treatment options and potential treatment outcomes.

**Conclusions:**

Serious games are a promising approach to health education and decision support for older men. Participants were receptive to the idea of a serious game as a decision aid in localized prostate cancer. However, usability issues are a major concern for this demographic, as is clarity and transparency of data sources.

## Introduction

The American Cancer Society predicts that 1 out of every 6 men will receive a diagnosis of prostate cancer in his lifetime, and for 1 in 35, this diagnosis will result in fatality [1]. Prostate cancer accounts for 25% of all cancer cases in American men [2]. The mortality rate from prostate cancer has decreased throughout the past decade, and diagnoses of early or local stage cancer have a 100% 5-year survival rate [2]. However, prostate cancer treatments often come with serious side effects, which can significantly affect patient quality of life in the short- and long-term.

### The Severe Uncertainty of Prostate Cancer Treatment

Men diagnosed with localized prostate cancer face a potentially life-altering decision with few facts to guide them. Clinical trials show no single best treatment [3]. While each of the widely accepted and mutually exclusive treatment options has a similar chance of extending life [3], risks of serious side effects such as incontinence and impotence differ according to procedure. Seeking a second opinion can add confusion to the decision-making process, as the majority of physicians recommend their own specialty’s treatment [4].

Additional factors influencing patients’ localized prostate cancer treatment decisions include fear and uncertainty, misconceptions about treatment efficacy and risks, and applying others’ experiences to their own cases [5]. Many men frequently choose an option simply to “get the decision off their minds” [6]. Research has shown that enhancing patient knowledge through education can significantly reduce feelings of uncertainty while simultaneously increasing confidence in decision making [7,8].

The uncertain task of choosing a treatment for localized prostate cancer is made more complicated due to comparable cancer-specific survival outcomes for all active treatments. In response to this added complication, the basis on which patients select a treatment has started to shift [9-14]. Focusing on health-related quality of life (HRQOL) can demonstrate measurable differences between treatment options in the short- and long-term. [15] As more published research reveals the HRQOL issues associated with prostate cancer treatment options [15,16], it is imperative that this research be made available to patients.

To date, a variety of decision aids have been developed to address the challenges of localized prostate cancer treatment decisions, and these decision aids have successfully demonstrated the ability to increase knowledge, enhance active involvement in decision making by patients, and decrease patients’ decisional anxiety [9,17]. Lin et al performed a meta-analysis of 13 studies examining the impact of decision aids on the experience of men diagnosed with prostate cancer, 7 of which included an assessment of decision aids’ effect on treatment choice [9]. Of these 13 studies, 4 in particular showed that decision aids can impact prostate cancer treatment choice (patients choosing a treatment other than surgery, a treatment that differed from their doctors’ recommendation, or changing their treatment choice from their initial preference) [10,17-19]. These studies have demonstrated the potential for decision aids to facilitate patient empowerment in the decision-making process, and have suggested an effective link to potentially more conservative treatment choices. However, more research is still needed on the real-world role of localized prostate cancer decision aids [9] and on whether decision aids actually help patients choose the treatment that best aligns with their lifestyle preferences [3,10].

### The Evolving Role of Serious Games

As the link between patients’ knowledge and positive health outcomes evolves, researchers increasingly look to interactive gaming technology as a vehicle for delivering health information. Serious games employ interactive game elements for purposes other than entertainment, such as education or training [20-22]. Lieberman defined an interactive game as an experience that involves rules, an assigned challenge that is serious in intent, movement toward a goal, and a defined ending [23]. Garris et al stated that games create a system that the user chooses to enter in order to accomplish a goal or overcome a problem contained within the game [24]. They also emphasized the iterative nature of games, that is, the built-in potential of the interactive game to support repeated “rounds” or game cycles.

Serious gaming represents a potential tool to effectively address health issues. Previous studies have shown success using games to impact factors such as disease management, behavior change, and health education [25,26]. While current literature focuses largely on the effect of serious health games on youth, evidence exists to support the use of serious health games to impact the health of older adults [27,28]. Approximately 40% of Americans aged 50 to 65 play video games [29]. Additionally, studies have shown that using interactive computer games can have a positive impact on elderly adults, specifically by improving levels of psychological health and cognitive functioning [28,30-32].

### Designing an Interactive Decision Aid

In response to the difficulties facing men diagnosed with localized prostate cancer, we decided to capitalize on the potential of serious games to assist with the treatment decision-making process. We sought to develop a serious game that would guide users through a simulated experience of the common impacts on HRQOL over the short- and long-term to help them determine which treatment strategy would be most acceptable to them, based on their personal preferences. As such, we based our serious interactive game, Time After Time, on the premise that eliciting users’ HRQOL preferences would offer unique and valuable insight to aid the decision-making process for localized prostate cancer treatment.

The game attempts to elicit user preferences regarding the impact of treatment side effects on a user’s preferred quality of life. Side effect scenarios are presented to users of Time After Time based on statistical probabilities derived from a large, prospective, multiregional study [15]. This study collected data from 1208 patients using the expanded prostate cancer index composite (EPIC), the landmark tool to measure HRQOL after prostate cancer treatment [33]. The EPIC survey tool evaluates patient function and bother in 5 major domains (vitality, urinary bother, urinary control, bowel control, and sexual function) at baseline and at 2, 12, and 24 months following treatment in order to characterize treatment-specific impacts on HRQOL. We used the published data set from this study [15] and the EPIC tool to functionally elicit user HRQOL preferences regarding side effects associated with the 3 active prostate cancer treatments (prostatectomy, brachytherapy, and external radiotherapy) and watchful waiting, accepted as standard care by the US National Institutes of Health National Cancer Institute [34]. Users of Time After Time rate side effects that they would potentially experience immediately after treatment, after 2 months, and after 12 months according to their personal lifestyle preferences.

Although EPIC was originally developed as a retrospective tool, it has been successfully employed in prospective HRQOL research. The developers of EPIC tested a prospective version of the survey instrument and found that the modified version accurately predicted urinary and bowel symptoms and was slightly less accurate at predicting sexual symptoms at 12 months posttreatment [35]. Pinkawa et al used the original, retrospective EPIC in a 2009 prospective study of the impact of age and comorbidities on HRQOL in localized prostate cancer and concluded that prospective use of EPIC was accurate across all domains from the patient’s perspective [36].

While EPIC has been validated as a prospective tool for predicting symptoms, our game attempts to elicit feelings about future events—a notoriously difficult task [37]. One recent study that prospectively examined how men ranked the importance of 11 factors in localized prostate cancer treatment found that men’s pretreatment feelings about what is important in prostate cancer treatment generally aligned with their posttreatment ratings [38]. While most men altered rankings of importance in at least 1 of the 11 factors 6 months after they chose their management course, the authors found that “the majority of pre-post evaluations were very consistent” [38].

The design of the game’s graphical interface used to present side effect combinations came from themes identified in an unpublished qualitative study of interviews with prostate cancer survivors in which men described the processes they went through to make their treatment decisions. A recurring theme that emerged throughout the interviews included a visual in which men laid out information regarding treatment options on a table as a key part of their decision-making process. We incorporated this visual into Time After Time by using playing cards to display potential side effects. These cards were laid out on the user’s screen, or virtual “table,” and organized by time period into the 5 main side effect domains used by EPIC survey.

### The Time After Time Experience

**Figure 1:**
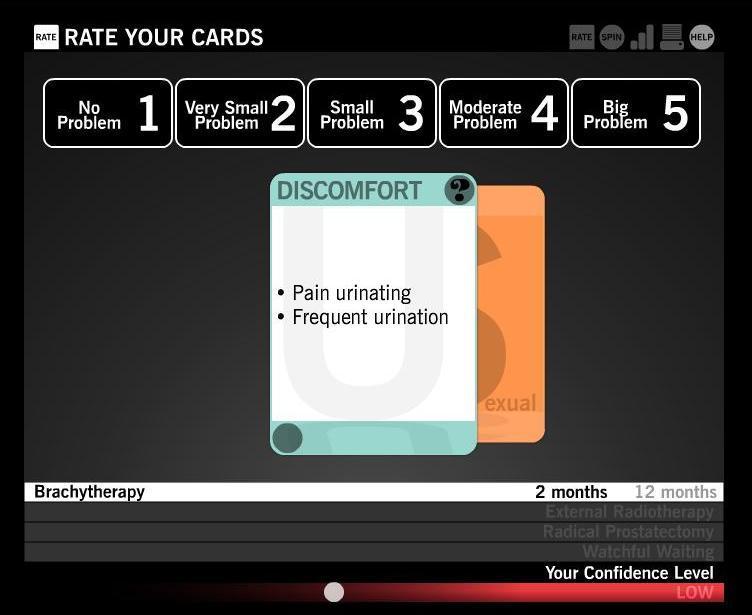
A side effect card in which side effects appear to the user using playing card imagery

**Figure 2:**
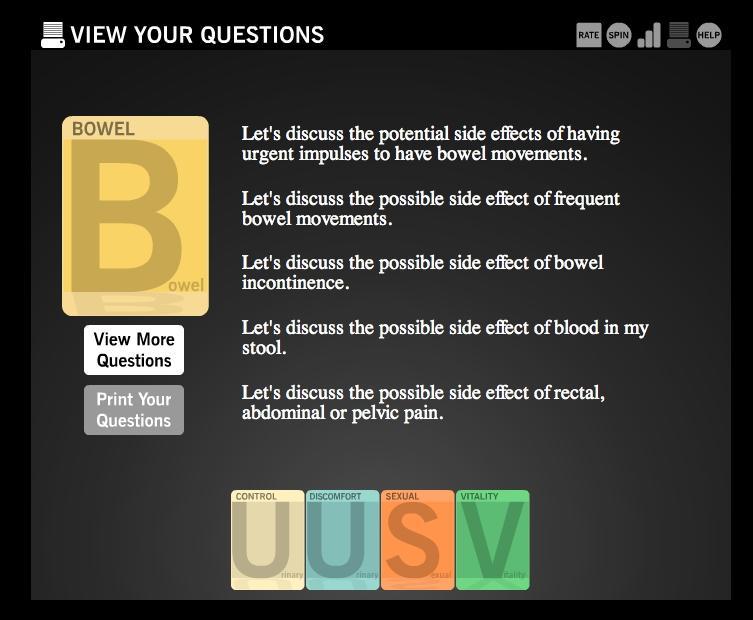
The section of the game that allows users to save questions that arise through interacting with the game for discussion with their care providers

Users begin playing Time After Time by securely logging in to the game using their personal username and password. Once they have logged in, they go through a guided orientation round before starting the official rounds of game play. White boxes appear with instructional explanations of imagery and symbols to help the user become familiar with the game’s interface and instruct their movements for each round to come. Time After Time allows the user to explore potential side effects of 4 treatment options: radical prostatectomy, brachytherapy, external radiotherapy, and watchful waiting. Side effects appear throughout the game using playing card imagery. For each treatment and each time period (immediately after treatment, 2 months after, and 12 months after), side effect card combinations are shown to the user (Figure 1). Each time a user reads a side effect card, he must rate it on a 5-point scale from 1, “no problem” to 5, “big problem.” Throughout this process, users may save any card that is unclear or that raises new questions to a list of questions for his doctor that can be accessed at the end of at least one round of game play (Figure 2).

**Figure 3:**
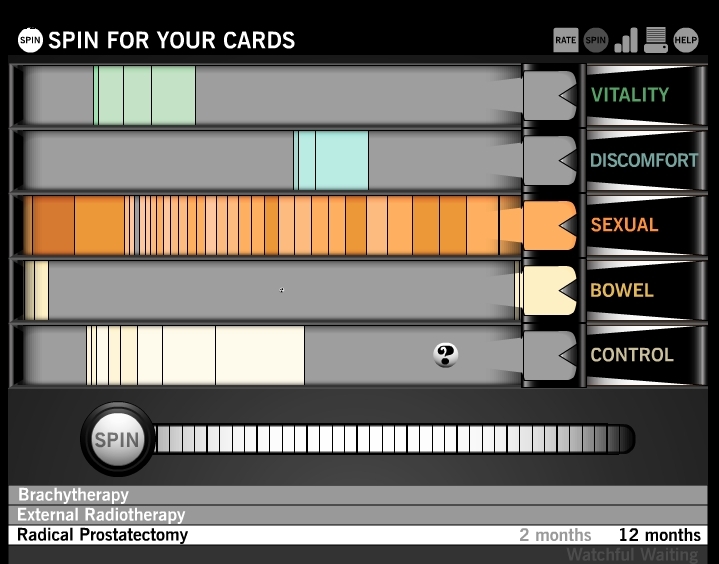
The spinner screen, which uses slot machine-like imagery to display probabilities of different side effects

Game play begins after the guided round by presenting the user with the immediate side effects of watchful waiting for him to rate according to his preferences. After watchful waiting, the user is similarly guided through the immediate effects of radical prostatectomy, brachytherapy, and external radiotherapy. After the user has rated the side effect cards in all 4 treatments in the time period immediately following treatment, he is introduced to the spinner screen using another guided orientation round. Using slot machine-like imagery, the spinner screen graphically displays probabilities of different side effects in 5 domains: vitality, urinary discomfort, sexual, bowel, and urinary control (Figure 3). Each time the user spins, the user is dealt side effect cards corresponding to the treatment and time period he is exploring. He then rates these cards using the same 5-point scale as during the immediately after treatment time period. The spinner and card imagery reinforce the role of chance in the sense that the cards the user is dealt are based on actual probabilities (derived from the EPIC studies) [15].

**Figure 4:**
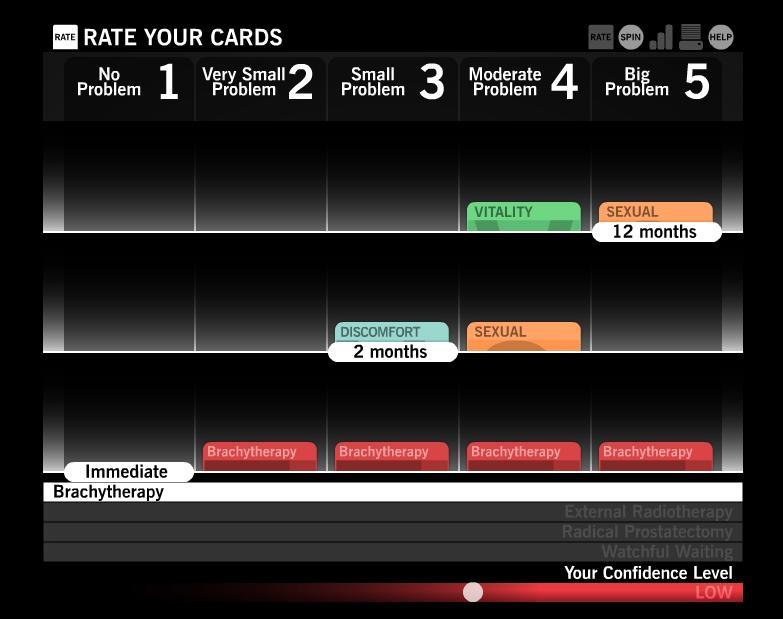
The summary screen, which displays the user’s rating of side effects by period and domain

**Figure 5:**
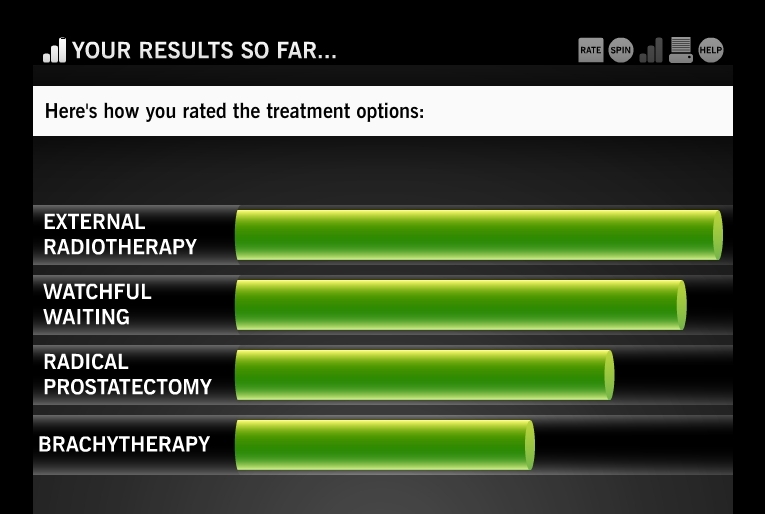
The first-round results screen, which includes a ranked comparison of all treatments (users are encouraged to complete more rounds to refine their results)

After the user has rated side effect cards at the time periods of 2 months posttreatment and 12 months posttreatment, he sees a categorized summary of how he rated cards by time period and side effect domain (Figure 4). This summary provides a visual cue of which domains he considers most and least problematic. Round 1 concludes when the user has completed rating the side effect cards in all of the treatments and time periods. After round 1, the user can view his results, which include the treatment for which the possible side effects best match the user’s preference ratings, a ranked comparison on all treatments (Figure 5), a ranked list of which domains concern the user most, and the list of questions generated for the doctor (Figure 2). The user can also play additional rounds of Time After Time to experience alternate side effect possibilities, thereby helping the user refine the game’s results to more accurately reflect their preferences.

Time After Time was designed to help patients understand how side effects could impact their HRQOL following different treatment options, with the goal of increasing patient confidence and empowering their participation in the decision-making process. This paper presents the results of a preliminary user-feedback study of Time After Time. A common practice in game design involves employing user input to inform various stages of game development [39,40]. Additionally, typical software development procedures require stages of testing, beginning with the earliest usable version of the technology, or the alpha version [41]. Following these practices, we recruited prostate cancer survivors to evaluate the alpha version of Time After Time*.* The study attempted to determine the acceptability and usability of the interactive decision aid for men diagnosed with localized prostate cancer and collect user feedback to inform future iterations of the serious game.

## Methods

### Study Design

The research team used a mixed methods approach to assess usability of Time After Time by combining a survey and focus group study. We focused on users early on and continuously in our development of the serious game, a practice commonly followed in game design [39-42]. The iterative development process we employed involved using quantitative and qualitative testing with small samples of potential users throughout the game development process to diagnose and address problems [41,43].

Prior to recruiting participants, the institutional review board at The Methodist Hospital Research Institute in Houston, Texas, approved the study protocol. A primary facilitator and 1 or 2 additional team members ran each focus group session. Facilitators consulted 2 experienced researchers with substantial experience in focus group mediation prior to the initiation of the study. Consultations included one-on-one instructional demonstrations with researchers and a review of literature on focus group facilitation [44]. A practice focus group session was held prior to recruiting study participants.

Focus group sessions consisted of a short introduction, game play, questionnaire completion, and group discussion. After 45 minutes of independent game play, participants completed a written survey based on their experiences. The unvalidated survey instrument was developed for this study with the goal of providing a quantitative assessment of the game’s usability and acceptability. The session ended with a 45-minute, semistructured focus group discussion based on a predetermined set of questions that guided topics covered by the groups.

### Participants

Inclusion criteria for focus group participants included men between the ages of 45 and 85 who were diagnosed with localized/early-stage (sometimes called stage I or stage II) [45] prostate cancer after 1998 and before November 2007. The age range was chosen to reflect the population of men typically screened for localized prostate cancer [46] and for whom the game is designed, as well as to maximize our ability to recruit participants for the study. Additionally, the range in dates specified for diagnosis ensured the exclusion of newly diagnosed patients in order to minimize any psychological risks that could arise by allowing an unvalidated version of the game to influence treatment decisions. We did not stratify by age, race, or socioeconomic class but did make efforts to include a diverse sample.

Recruiting for focus groups took place during monthly meetings of a local Houston prostate cancer support group, as well as through emails to the groups’ online listserv. During the 6-month study period, a total of 13 participants attended 1 of 4 focus group sessions (3 groups of 3 participants and 1 group of 4 participants). Recruiting was concluded following the completion of these 4 focus group sessions, as focus group transcript analyses revealed a repetition of themes and responses indicating we had achieved a level of saturation appropriate for the preliminary testing of Time After Time [43,47].

### Data Collection and Analysis

We collected quantitative measures of acceptance and usability from an 18-item instrument based on a 7-point Likert scale (Multimedia Appendix 1). This instrument was developed in line with user-centered game design principles, which use surveys or questionnaires to collect attitudinal data regarding participant views [41]. In keeping with this methodology, Likert items were designed for participants to rate their overall impressions of the game, how easily they were able to use the game, and usability of specific game features.

Focus group discussions were recorded and transcribed verbatim. Using grounded theory as the basis for analysis of focus group data [48], an audit committee of 5 researchers conducted a thorough review of the focus group session transcripts. Of the 5 researchers, 2 were experienced in coding and had training in qualitative analysis methods. Researchers induced thematic patterns from their analysis of the transcripts and, as such, were able to define and report frequencies of key themes. Definitions were arrived upon through committee discussions and dialogue, and disagreements were resolved through reliance upon the verbatim transcripts to ensure the highest level of consistency and accuracy.

Specific attention was paid to the identification of themes regarding acceptability and usability of Time After Time. Acceptability was defined as participants’ willingness to use Time After Time specifically, and an interactive computer game in general, in decision making for localized prostate cancer treatment. Researchers defined usability as the potential user’s ability to navigate through a session of game play. The key measure of usability was the users’ self-reported perceptions of the game’s ease of use [43].

## Results

The results of the focus groups address the study’s 3 major questions: (1) Do users accept the interactive computer game as a decision aid for localized prostate cancer? (2) Can users easily navigate and use the interactive computer game? (3) Does the game effectively increase users’ confidence and participation in the decision-making process?

### Survey Instrument Results

Participants completed the survey instrument immediately following game use (Multimedia Appendix 2). Answers were rated on a scale of 1 to 7, 1 corresponding to “strongly agree” and 7 corresponding to “strongly disagree.” The scale was designed so that means closer to 1 would indicate a more positive response and means closer to 7 would indicate a more negative response. Likert data were analyzed using mean values, which is in accordance with standard assumptions for interval data analysis [49].

Item 4 of the survey (“An interactive website is effective for providing information”) had the mean closest to 1, indicating positive perception of an interactive computer game’s ability to provide information on treatments and side effects (mean 2.77). Survey results for item 8 of the instrument (“While using the interactive website, it is clear which time period is being explored”) indicated participants generally understood the game’s simulated time period of immediately, 2 months, and 12 months after treatment, but the proximity to the middle point suggests that this area of simulation could be improved in the future (mean 3.08). Features that received negative user feedback included the “spinner” screen displaying side effect probabilities (mean 4.67), and the screen displaying the final treatment ranked highest by the user (mean 4.92).

### Acceptability: The Game’s Role in the Treatment Decision Process

Focus group discussions began with the participants describing their own treatment decision-making process. All participants stated that they conducted their own research when trying to decide on a treatment for localized prostate cancer. Speaking one-on-one with doctors, friends, or family members previously diagnosed and treated for prostate cancer was cited by 10 out of the 13 participants as crucial to their personal research on treatments. In addition, the men revealed that in conducting their own research on localized prostate cancer and its treatment options, they often used a diary or journal for personal note taking. Participants discussed using such notes during appointments with their doctors and stressed the importance of recording their own notes and questions throughout their decision-making process.

The utility of Time After Time’s feature allowing users to highlight side effect cards they do not understand and print questions at the end of the game was validated by participants in all focus groups. Participants repeatedly expressed their appreciation for the game’s option to print questions on treatments and side effects, given their tendency to use personal note taking to document their research and inform conversations with their doctors. One user stated,

I think [the print option is] good because to me…because so many times you go to the doctor and you’re just so overwhelmed. And you don’t know what to ask them, because you say…well, the doctor says, “Do you have any other questions?” and I say, “Well, I can’t remember what they are.”

### Acceptability: Users’ Perceptions of a Serious Game for Localized Prostate Cancer

Participants were asked to discuss their feelings surrounding the use of an interactive computer-based decision aid in general, and Time After Time in particular, to make a treatment decision for localized prostate cancer. In all, 5 men stated they would not use the Time After Time computer game as a way to definitively choose a course of treatment. However, when asked whether they would use the game as a part of their decision-making process, 10 of the 13 participants reported that they would welcome it as a mechanism to enhance their education, in addition to their other preferred methods of research. As one man stated,

I look at [Time After Time] as being a tool, one of the tools, not the final tool. I don’t think I would make a decision based on this, but I would use it and then use other things to [help me] make a decision.

Participants found usefulness in the game’s ability to raise more and better questions for their doctors, as well as its ability to reveal new information on the side effects of treatment they should consider during their decision making process. Commenting on the lack of understandable information when researching his own prostate cancer treatment options, one participant expressed a desire for “a tool that might help you make a better decision…because I was ravenous for information when I found out about [my diagnosis].”

### Usability: Game Design and Content

The focus group participants revealed flaws in the game design that sometimes made it difficult for them to navigate and sometimes distracted them from the game’s intended purpose. Participants often had trouble completing a full round of the game without help from study staff and were not always clear on what they needed to do next in the game. However, the majority of men verbally reported successfully grasping the concept of treatment simulation for 3 time periods.

Another aspect of treatment decision making that participants discussed was the descriptions of the side effects covered throughout the game. A third of the participants requested greater detail in the descriptions on the side effect cards. For them, phrases like “urinary incontinence” or “erectile dysfunction” did not communicate the actual experience or meaning of the side effect for someone who lacked personal experience. Additionally, about 30% of participants requested that the game include a wider variety of treatments that extended beyond the 4 options presently included. Rapidly developing technologies and the emergence of new treatment methods represented important considerations for men in the midst of the decision-making process.

### Usability: Time After Time and Decision Making in Localized Prostate Cancer

Focus group discussions revealed that the game’s design currently leaves out several aspects of treatment decision making that participants identified as crucial features of the experience. For example, many participants reported that when they made their own treatment decisions, long-term survival rate was the most important factor they considered. The possibility of cancer recurrence, as related to specific treatments, represented a vital aspect of their treatment decision. Thus, participants expressed a desire for the game to cover a time period of 5 to 10 years, as opposed to just 12 months, to reflect the possibility of recurrence posttreatment. Also, participants requested that the game more effectively communicate the possibility for side effects to dissipate over time. Many brought up the availability of surgery or medication, which may have the ability to diminish the severity or eliminate negative side effects in the long term. As such, participants requested that the game include a reference to the variety of options available posttreatment to prostate cancer survivors.

A dominant theme brought up in all focus groups was the request for the inclusion of a prologue introducing Time After Time that contains more in-depth explanations regarding the goal of the game, limitations of the analysis process, and the context in which men diagnosed with localized prostate cancer should use Time After Time. Additionally, 10 out of 13 participants expressed the desire for Time After Time to include user input. As one participant described,

For some people, it’s about “man, I’m going to live as long as I can.” I know people like that. I’m not one of those people. I want to live a really good life for whatever time I have left. And those sorts of qualifying questions about “where are you in life?” [are what is missing from Time After Time]…[What the game needs] is a little bit more qualification about who are you and what are you doing.

A recurring theme in focus group sessions surrounded the availability of the medical data on which Time After Time was based. Over half of the participants requested a transparent description of the statistical foundation supporting the side effect scenarios generated while using the game. Of the 13 participants, 8 wanted the game to display the numerical probabilities corresponding to side effect scenarios and final treatment rankings produced by the game. All of the men expressed a desire for increased transparency in describing how the game used their feedback on the side effects to produce the results they received. However, 7 men did not recognize the connection between the side effect scenarios presented and the probability statistics on which the game was based.

In summary, the results of the focus groups revealed a role for an interactive computer game such as Time After Time in the decision-making process for localized prostate cancer, provided that future iterations address specific usability issues (navigation and introductory information), content issues (longer time frames, extended descriptions of treatment, and posttreatment options), and acceptability issues (personalization and direct explanations of statistical data).

## Discussion

Patients diagnosed with localized prostate cancer must choose among a range of treatment options, most commonly watchful waiting, radical prostatectomy, external beam radiotherapy, and brachytherapy. However, selecting the best treatment presents patients with a significant challenge due to the lack of evidence identifying a single option as most effective for treating localized prostate cancer. Patients must consider not only the survival consequences and acute morbidity of each approach to treatment, but also the possible effects those approaches can have on quality of life. The difficulty involved in choosing a treatment plan for localized prostate cancer makes the availability of accurate, accessible, and understandable information crucial to the treatment decision process. Our findings support the use of serious video games as a potential way to enhance education on treatment side effects and prepare patients for more active participation in conversations with their medical team.

### Bringing Side Effects Into Focus

The majority of participants named survival and chance of recurrence as primary factors impacting their treatment decision. The men exhibited the tendency for patients to neglect how side effects of treatments could affect their HRQOL in their decision-making process. The gap in adequate and accurate knowledge regarding side effects associated with localized prostate cancer treatments presents an opportunity and a need for enhanced patient education.

In the focus group sessions, participants validated the game’s ability to focus their attention on the side effects of prostate cancer treatments. This new focus helped them differentiate between treatment choices and view the possible outcomes of each treatment in light of their lifestyle preferences. As one participant said,

[Time After Time brings] side effects right up front as part of decision making because you know even though we don’t think that things like incontinence or impotency would affect you as much as cancer—if you are comparing cancer to everything else, cancer is going to win—but prostate cancer is not that way.

As an interactive decision aid, Time After Time can fill this knowledge gap by providing men diagnosed with localized prostate cancer with evidence-based education on the HRQOL impacts of treatment side effects.

### The Need for Personalization in Game Design

One of the most common remarks made by participants surrounded the absence of game personalization through the input of user-specific data. Personal life situations and lifestyle preferences represented crucial components of every focus group participant’s self-described treatment decision process. In all focus groups, men highlighted the crucial need for the game to address factors such as age, marital status, and physical health before diagnosis. For them, the game must have a way of incorporating personalized information into its analysis.

The results of the survey instrument served to support the overarching themes and purpose of the Time After Time interactive game (Multimedia Appendix 2). Participants responded more positively (as indicated by means closer to 1) to items regarding the overall idea of the game and its ability to provide users with information on treatments and side effects. Negative results (indicated by means closer to 7) focused on specific implementation of game features. We believe that the participants’ generally positive attitudes toward the idea of the game for the general public, combined with less positive responses about the applicability of the game to their own personal cases, reflect their expressed desire for more personalization. Participants could imagine the game being a valuable tool for a patient who fit the “norm” and/or who has yet to explore the nuances of the treatment decision, but they personally found aspects of their own situations that they would have liked the game to address in a transparent way.

### Designing Serious Video Games for Men With Localized Prostate Cancer 

While participants validated the utility of the option to print out questions for their doctors regarding treatments and side effects in the current version of the game, future iterations of the game should incorporate a more interactive note-taking feature. Enhancing patients’ ability to record relevant information, thoughts, or questions could significantly improve their experience of the game as a decision aid. Additionally, participants emphasized the immense value they found in conversations with other survivors, family members, and doctors during their decision-making process. Adding a social networking component to Time After Time could enhance its relevancy by integrating the benefits of person-to-person communication with the game’s educational value.

Participants also wanted the game to include the actual probability data related to each side effect domain displayed as a number and in some kind of chart or graph in addition to the more interpretive format in which the data are currently presented. A total of 9 men expressed a desire for such numbers accompanied by background data to fully grasp the concept of probability demonstrated through the presentation of side effect scenarios. For them, Time After Time must include these data and an enhanced level of detail on the game’s foundational concept to establish the overall purpose and validate the credibility of the game as a decision aid.

A review of the focus groups supports Time After Time’s potential to provide useful and relevant education on treatment side effects and to augment patient conversations about treatment options with their doctors. However, a need for a more literal translation of game concepts became evident in participants’ difficulties interpreting instructions, navigating different user modes and screens, and understanding the correlation between abstract themes of probability and side effect scenarios. Incorporating a more direct translation of the game’s goals and functional steps could greatly enhance Time After Time’s ability to act as a supplement to current methodologies used in localized prostate cancer decision making.

### Limitations

Some limitations affecting this study may have had an impact on the range and quality of information gathered from the focus groups and survey instrument. The small sample size recruited solely from an active prostate cancer support group and its online community granted us valuable insight into how men diagnosed with localized prostate cancer experienced treatment decisions but leaves this study unable to make a wider generalization to all men diagnosed with the disease. Future evaluations of Time After Time should strive to recruit participants from sources other than support groups to reduce potential recruiting bias that may arise from men in support groups having different needs than the population of men with localized prostate cancer regarding the treatment decision-making process. Additionally, our use of participants who had already received treatment for localized prostate cancer may also represent a limitation. Although the questions were worded to minimize this factor, the participants’ perspectives as men who have lived through the treatment decision are likely to differ from the perspectives of the game’s intended audience, that is, men who have not yet chosen a treatment.

The presence of a female discussion facilitator in each of the focus group sessions presents a potential limitation to the type and extent of responses given during focus group discussions, as participants may have felt reluctant or anxious discussing sensitive side effect issues with a female. An introductory explanation of the game given by the facilitator to familiarize participants with the game may have resulted in a skewed participant experience.

Predicting how one will feel in the future, particularly about novel experiences such as surgery, represents an inherently difficult task [37,50]. While several studies have addressed decisional regret in localized prostate cancer treatment [51-53], we were able to find only one that prospectively examined whether expectations men had about how they would feel about specific aspects of management matched their lived experiences posttreatment [38]. Although this study’s results were promising, people’s ability to predict their feelings regarding future health states remains an important limitation of our study. Future projections of preferences regarding conditions not yet experienced are often subject to biases that can skew the accuracy of people’s predictions [37]. In response to this issue, other researchers in our group have completed preliminary validation studies on a prospective tool for evaluating health-related quality of life based upon the well-accepted Medical Outcomes Study SF-36 Health Survey [54-56].

Finally, there are limitations to the use of Likert-type self-report scales. Self-report questionnaires may measure self-concepts that do not necessarily reflect actual behavior and may be subject to bias and error. Another limitation of Likert scales is the primacy effect [57,58], where respondents are more likely to choose the options on the left side of the page. (In this study, the negative options were on the right side of the page.) However, the mixed methods approach used combined observation of participants and open-ended discussion of questions with a scaled survey, which has been suggested as a tool for partially overcoming these limitations [59].

### Conclusion

Our initial research has made clear that game-based interactive decision aids for localized prostate cancer like Time After Time have the potential to fill an important need for newly diagnosed patients. The majority of the study participants believed that Time After Time represents a valuable step in the development of an appropriate decision tool for localized prostate cancer. Participants verified that the game meets the goals of increasing focus on HRQOL issues, generating questions for the patient’s health care team, and providing a new educational avenue to augment the patients’ participation in choosing a treatment for localized prostate cancer.

However, opportunities to improve the game’s usability exist. For the subsequent version of the game, researchers will attempt to take further steps in improving the standard of decision making for localized prostate cancer. We plan to modify and enhance the design and functionality of Time After Time to provide a construct through which patients can match their expectations and preferences with realistic goals, thereby better preparing them for the outcomes of their treatment choices and reducing the decisional conflict typically associated with localized prostate cancer.

## Multimedia Appendix 1

Likert survey instrument used to assess acceptance and usability of interactive computer games

## Multimedia Appendix 2

Graph of survey instrument results

## Abbreviations

EPIC: expanded prostate cancer index composite
HRQOL: health-related quality of life
