# Supplementary material for: Assessing the Acceptability and Usability of an Interactive Serious Game in Aiding Treatment Decisions for Patients with Localized Prostate Cancer
Source: J Med Internet Res. 2011 Jan 12;13(1):e4. doi: 10.2196/jmir.1519 (PMC3221354; doi:10.2196/jmir.1519)
Supplement: Supplementary file 1 [file jmir_v13i1e4_app1.pdf]

Multimedia Appendix 1: Survey Instrument

Please rank the following statements according to what you agree with. There are no right or wrong answers. Please circle the number corresponding to how you feel about a statement.

	**Strongly Agree**						**Strongly Disagree**
1. If it had been available at the time of my diagnosis, the interactive website would have improved my confidence.	1	2	3	4	5	6	7
2. The interactive website is useful.	1	2	3	4	5	6	7
3. At the time of my diagnosis, the game would have increased my knowledge.	1	2	3	4	5	6	7
4. An interactive website is effective for providing information.	1	2	3	4	5	6	7
5. The instructions are sufficient.	1	2	3	4	5	6	7
6. The *tone* of the interactive website is appropriate.	1	2	3	4	5	6	7
7. While using the interactive website, it is clear which treatment is being explored.	1	2	3	4	5	6	7
8. While using the interactive website, it is clear which time period is being explored.	1	2	3	4	5	6	7

9. While using the interactive website, the screen on Handout 1 is useful.	1	2	3	4	5	6	7
10. While using the interactive website, the screen on Handout 2 is useful.	1	2	3	4	5	6	7
11. The screen on Handout 3 is useful.	1	2	3	4	5	6	7
12. If it had been available at the time of my diagnosis, Handout 3 screen would have affected my final decision.	1	2	3	4	5	6	7
13. The screen on Handout 4 is useful.	1	2	3	4	5	6	7
14. The screen on Handout 5 is useful.	1	2	3	4	5	6	7
15. The screen on Handout 6 is useful.	1	2	3	4	5	6	7
16. Using the interactive website would help me discuss treatment options with my doctor.	1	2	3	4	5	6	7
17. If it had been available at the time of my diagnosis, I would have felt comfortable using this interactive website to aid in my decision-making.	1	2	3	4	5	6	7
18. I would recommend this interactive website to a friend who has just been diagnosed with prostate cancer.	1	2	3	4	5	6	7
