# Supplementary material for: Assessing the Acceptability and Usability of an Interactive Serious Game in Aiding Treatment Decisions for Patients with Localized Prostate Cancer
Source: J Med Internet Res. 2011 Jan 12;13(1):e4. doi: 10.2196/jmir.1519 (PMC3221354; doi:10.2196/jmir.1519)
Supplement: Supplementary file 2 [file jmir_v13i1e4_app2.pdf]

Multimedia Appendix 2

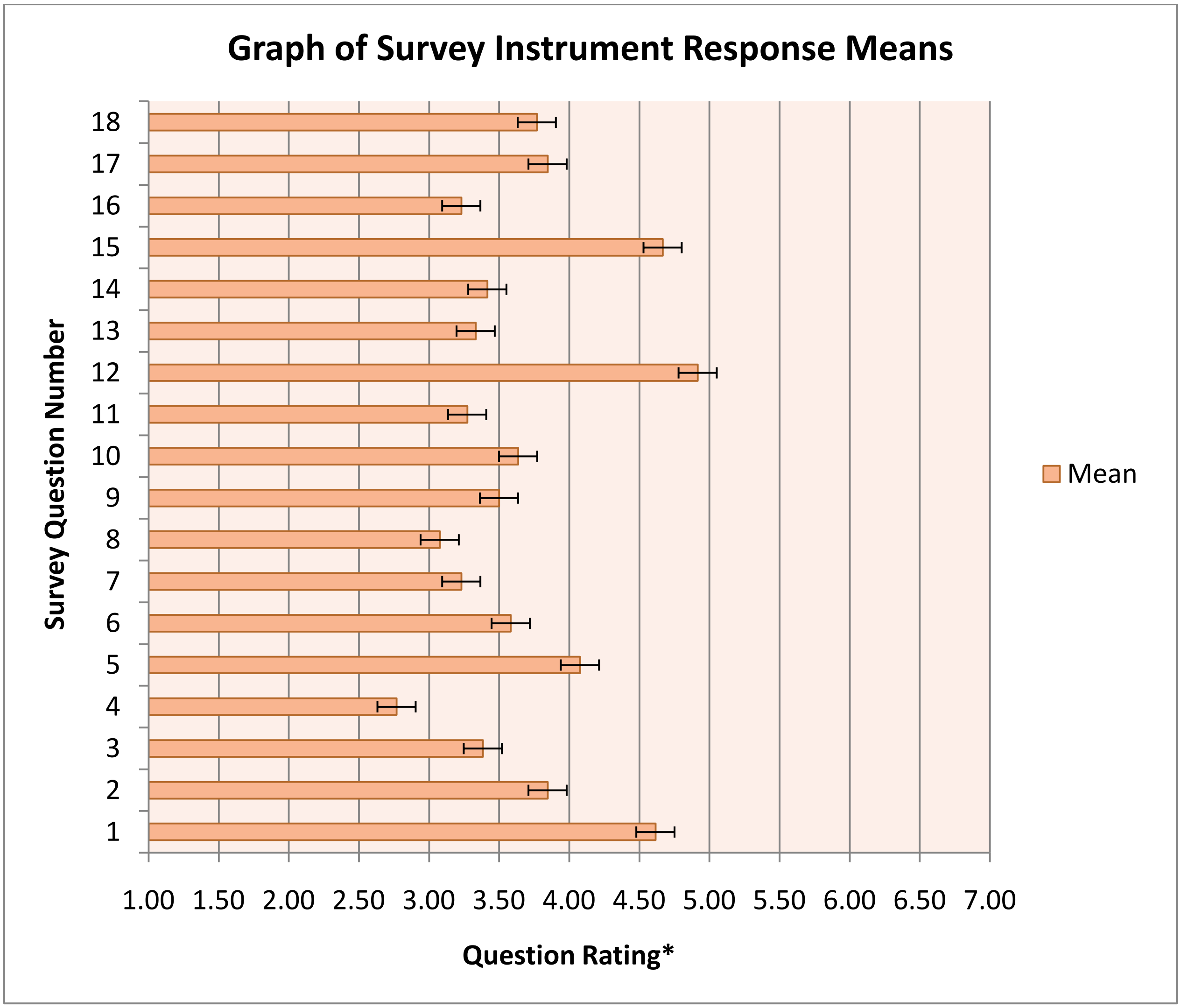

*Participants rated questions on a Likert scale of 1 – 7, with a rating of 1 corresponding with “Strongly Agree” and a rating of 7 corresponding with “Strongly Disagree.”
